# Commencement

**Published:** 1878-04

**Authors:** 


### COMMENCEMENT.

The thirty-second annual commencement of the Ohio College of Dental Surgery was held on the evening of March 6th in the hall of the college.

The exercises were commenced by reading the scriptures and prayer, by Rev. Jas. Chester, when, after some appropriate remarks, the degrees were conferred by Dr. Jas. Taylor, President of the Board of Trustees, after which he delivered his address of counsel to the graduates.

The degree of D. D. S. was conferred upon Chas. H. Rosenthal, of Indiana; Wm. H. Creighton, of Ohio; M. P. Walsh, of Illinois; Thos. A. Stephenson, of Ohio; Chas. Fithean, of Kentucky, and W. D. Phillips, of Ohio.

Dr. A. O. Rawles, of Kentucky, delivered the annual address which was heard with great interest. The reply on behalf of the class was made by Dr. Thos. A. Stephonson, of Cincinnati.

The exercises were concluded by the distribution of two prizes, one to the senior class for highest attainments in operative dentistry, this was awarded to C. H. Rosenthal, and the other to the junior class for best attainments in mechanical dentistry was awarded to Wm. Moffit, of Kentucky.

Lest the giving of this prize to this member of the senior class might lead to wrong conclusions in reference to the other members of the class, it is proper to say that the formal examination to which they even subjected was a rigid one, and occupied about sixteen hours, wholly written, and the average per cent. of correct answers was above ninety, and none below eighty, and practicable operations and treatment were presented by each member of the class, all of which was pronounced very satisfactory.

